# Bridging the monitoring gap in resource-limited settings: A head-to-head evaluation of two Chinese HIV-1 RNA quantification assays and Roche TaqMan

**DOI:** 10.1371/journal.pone.0354737

**Published:** 2026-08-05

**Authors:** Jiafeng Zhang, Xiaobei Ding, Qin Fan, Jiezhe Yang, Yan Xia, Chengliang Chai

**Affiliations:** 1 Department of HIV/AIDS Control and Prevention, Zhejiang Provincial Center for Disease Control and Prevention, Hangzhou, Zhejiang, China; 2 Key Lab of Vaccine, Prevention and Control of Infectious Disease of Zhejiang Province, Zhejiang Provincial Center for Disease Control and Prevention, Hangzhou, Zhejiang, China; 3 Zhejiang AIDS/STD Prevention Association, Hangzhou, Zhejiang, China; World Health Organization, SWITZERLAND

## Abstract

**Background:**

HIV-1 RNA quantification assays are essential for evaluating antiretroviral therapy (ART) efficacy and diagnosing HIV infection, playing a pivotal role in HIV/AIDS management and treatment.

**Methods:**

This study compares two Chinese-manufactured HIV-1 RNA quantification assays (Livzon, SymBio) with the Roche COBAS AmpliPrep/COBAS TaqMan HIV-1 Test v2.0 (reference assay) using plasma samples from 279 HIV-infected individuals.

**Results:**

The reference assay had a positive detection rate of 92.47% (258/279), while Livzon and SymBio assays both achieved 94.62% (264/279). The positive agreement was 98.06% (95% CI: 95.54–99.17%), and the overall agreement was 94.27% (95% CI: 90.89–96.44%). In the linear regression analysis, Livzon demonstrated a correlation coefficient (r) of 0.989, and Bland–Altman analysis indicated a mean bias of 0.18 log10 copies/mL; SymBio showed r = 0.988 with a mean bias of −0.09 log10 copies/mL. Both assays showed Pearson correlation coefficients greater than 0.985 and biases ranging from −0.17 to 0.24 log10 copies/mL compared with Roche across diverse HIV subtypes.

Direct comparison between the two domestic assays revealed a strong correlation (r = 0.988), with a mean difference (Livzon – SymBio) of 0.27 log10 copies/mL. Regarding cost and turnaround time (TAT), the domestic assays were priced more than 30% lower, and batch TAT was reduced by 20–50% relative to the reference assay.

**Conclusions:**

Both Chinese-manufactured assays demonstrated strong quantitative correlation, and consistency, meeting the performance standards required for HIV-1 viral load detection. These assays also offer substantial cost and turnaround time advantages, making them well-suited to bridge the HIV-1 monitoring gap in resource-limited settings.

## Introduction

The global burden of HIV/AIDS remains a significant challenge, with a substantial number of individuals affected by HIV worldwide. According to WHO data, approximately 39.9 million people were living with HIV/AIDS (PLWHA) globally in 2023 [1], with considerable efforts being made to expand access to antiviral treatment (ART) and care. In China, ART coverage has expanded steadily, reaching approximately 1.22 million people by the end of 2023.

HIV viral load (VL) testing provides essential insights into the replication dynamics of the virus within the host and plays a crucial role in evaluating ART effectiveness, treatment response, and disease progression. Monitoring VL dynamics guides timely treatment adjustments, thereby optimizing clinical management and patient outcomes. In 2013, the WHO recommended VL testing as the preferred method to diagnose and confirm ART failure [2]. Furthermore, VL serves as a proxy measure to assess the risk of transmission and evaluate the efficacy of preventative measures at both individual and population levels [2].

National guidelines emphasize the critical importance of VL testing and its expansion for treatment monitoring. The Chinese “Manual of Free Antiviral Drugs for HIV/AIDS Treatment” (5th Edition) [3] recommends VL testing at baseline, 6 months, 12 months, and at least once a year thereafter. The “Guidelines for the Diagnosis and Treatment of AIDS in China” (2024 edition) [4] recommend VL testing at baseline, 4 − 8 weeks after treatment, and then every 8 − 12 weeks until complete virological suppression is achieved. Similarly, the “Guidelines for the Use of Antiretroviral Agents in HIV-1-Infected Adults and Adolescents” (2023 Update) [5] in the USA recommend conducting VL testing within 2–4 weeks of initiating ART, followed by regular testing every 4–8 weeks until viral suppression is achieved. Subsequent VL testing is advised every 3–4 months during the first two years of treatment to monitor treatment response and disease progression effectively [5].

In 2022, nearly 29 million VL tests were estimated to have been conducted in low- and middle-income countries (LMICs) [2]. As the volume of VL tests continues to increase, associated costs are also rising, placing a significant burden on national budgets. WHO emphasizes the need for alternative, cost-effective assays to sustain viral load monitoring [6]. The Clinton Health Access Initiative (CHAI) 2025 HIV Market Report underscores the critical need for cost-effective diagnostic alternatives in LMICs due to declining international funding [7]. Historically, HIV VL testing platforms and reagents in China were almost entirely imported, and their high costs have substantially hindered the scale-up of testing frequency and coverage, particularly in resource-constrained regions. Therefore, there is an urgent need for domestically produced HIV-1 VL assays that meet international performance standards while remaining cost-effective. This would support the ongoing efforts in HIV/AIDS prevention and management. In recent years, several domestically produced HIV VL assays have been successfully developed and marketed in China, representing significant progress in this area. Therefore, validating these domestically developed assays against established global standards is essential for their wider implementation.

The present study conducted a head-to-head, real-world clinical verification of two commercially available, National Medical Products Administration (NMPA)-approved Chinese HIV-1 VL assays alongside the Roche COBAS AmpliPrep/COBAS TaqMan HIV-1 Test v2.0 (reference assay). Rather than repeating the foundational analytical validation previously completed for regulatory registration, this comprehensive assessment was designed to verify the performance characteristics and clinical utility of these approved assays in a real-world diagnostic setting, thereby supporting their pivotal role in optimizing treatment regimens and public health interventions.

## Materials and methods

### Clinical samples

Sample size was determined based on the Clinical and Laboratory Standards Institute (CLSI) EP09-A3 guidelines for method comparison. The guideline recommends at least 100 patient samples for manufacturer-conducted validation studies to establish bias claims across the entire measuring interval. To ensure sufficient coverage of diverse HIV subtypes and viral load ranges, including the critical low-level viremia range, we enrolled 279 participants between May 2023 and November 2023 in this study. The inclusion criteria were as follows: (1) newly diagnosed HIV/AIDS patients without antiviral therapy; (2) patients with short-term antiviral treatment (no more than 3 months); and (3) patients with incomplete viral suppression (detectable VL or ≥20 copies/mL) after treatment. Patients meeting any of the above criteria were eligible for inclusion in the study. Ten mL of whole blood was collected from each participant using ethylenediaminetetraacetic acid (EDTA) as an anticoagulant after written consent was obtained. The whole blood samples were centrifuged at 3000*g* for 10 minutes, and the plasma was aliquoted into storage tubes and stored at −80°C until further testing. To prevent HIV-1 RNA degradation, plasma separation and preservation were completed within 6 hours. This study was approved by the Medical Ethics Committee of the Zhejiang Provincial Center for Disease Control and Prevention (2022-048-01) and was conducted in accordance with the Declaration of Helsinki, following all approved guidelines and regulations.

### HIV viral load (VL) assay

Samples were stored at –80°C without thawing until analysis. To ensure uniformity, aliquots from the same sample were thoroughly thawed and homogenized in a 5 mL cryotube before testing. Three HIV-1 VL assays were performed in parallel following the manufacturer’s instructions. All assays were initiated within 1 hour of each other to minimize pre-analytical variation.

### The two evaluated HIV-1 VL assays

Both the Livzon and SymBio assays are NMPA-certified in vitro diagnostic (IVD) systems designed for the quantitative detection of HIV-1 RNA, with their Limit of Detection (LoD) established using the WHO 4th International Standard (NIBSC code: 16/194). These assays were utilized in the current study according to the manufacturers’ instructions for routine clinical testing.

The Livzon HIV-1 VL assay (Livzon Diagnostics Inc., Zhuhai, China; NMPA approval No. 20193400849) featured single-dose lyophilized reagents that contain all the components needed for HIV-1 RNA quantification. The target genes were *pol* and *LTR*, which were amplified using the internal standard method. According to the manufacturer’s instructions, 0.65 ml of plasma was required, and the test had a linear quantitative range from 17.5 to 5.8 × 10^7^ copies/mL with a limit of detection (LoD) of 17.5 copies/mL (1 copy = 1.71 IU). The equipment used for this assay included a Microlab^®^ STARlet automated liquid handler (Hamilton Company, Reno, Nevada, USA), which completes RNA extraction and configures the PCR system automatically, and a SLAN-96P real-time PCR instrument (Hongshi Medical Technology Co., Ltd., Shanghai, China).

The SymBio HIV-1 VL assay (SymBio Life Science Co., Ltd., Suzhou, China; NMPA approval No. 20213400470) was performed on a Pre-NAT II instrument (SymBio Life Science Co., Ltd., Suzhou, China), which automates RNA extraction and PCR setup, followed by detection on the SLAN-96P real-time PCR instrument. The input plasma sample volume was 0.60 ml, and the LoD was 19.3 copies/mL (33 IU/mL). HIV-1 RNA quantification was based on standard curves generated from five different concentrations of external standards. The linear quantitative range was 29.2 copies/mL (50 IU/mL) to 1.2 × 10^7^ copies/mL. The assay utilized a dual-target design against highly conserved regions of the *gag* and *pol* genes.

### Reference HIV-1 VL assay

The Roche COBAS HIV-1 VL assay (COBAS^®^ AmpliPrep/COBAS^®^ TaqMan^®^ HIV-1 test, version 2.0) was used as a reference method and was performed on a Roche COBAS^®^ AmpliPrep instrument and COBAS^®^ TaqMan^®^ 48 Analyzer (CAP/CTM) (Roche Molecular Diagnostics, Pleasanton, CA, USA). This platform was selected because it remains the most widely utilized and recognized “gold standard” for HIV-1 nucleic acid testing across both clinical hospitals and the Centers for Disease Control and Prevention (CDC) systems in China. Utilizing this established infrastructure ensures that our findings reflect real-world implementation conditions and provide practical guidance for current diagnostic settings. The input volume of plasma was 1.00 ml, with an LoD of 20 copies/mL. The Roche assay detected both the *gag* and *LTR* genes using the internal standard method. The quantitative linear range was 20 to 1.0 × 10^7^ copies/mL (1 copy = 1.71 IU).

### HIV genotyping

The protease gene and the first 299 residues of the reverse transcriptase gene (1316 bp, HXB2: 2147-3462) were amplified by reverse transcriptase‒polymerase chain reaction (RT‒PCR) and subsequent nested PCR [8]. Two methods were employed to identify the subtypes: the online automated HIV-1 subtyping tool COMET HIV-1 [9] (https://comet.lih.lu/index.php?cat=hiv1) and phylogenetic analyses via MEGA v6.0 software. The neighbor-joining method was used in the phylogenetic analyses for genotyping. Reference sequences covering the major HIV-1 subtypes and CRFs were obtained from the Los Alamos National Laboratory HIV sequence database (https://www.hiv.lanl.gov). The Recombination Identification Program (RIP) v3.0 [10] (http://hiv-web.lanl.gov) was used to analyze potential intersubtype recombinations. In cases of discordant subtype assignment, the results from the phylogenetic analysis (MEGA v6.0) were considered definitive, serving as the gold standard over the automated COMET tool.

### Cost and turnaround time (TAT) evaluation

In addition to analytical performance, operational data regarding reagent costs and turnaround time (TAT) for the three assays were collected. For cost evaluation, the list prices of domestic assays (Livzon and SymBio) and the Roche COBAS HIV-1 VL assay were compared, with particular attention to the relative price differences. For TAT assessment, the workflow time required to complete a 96-test run (including internal controls) was measured from the start of automated RNA extraction to the availability of final results. Pre-analytical processes, such as instrument start-up, shutdown, and manual pipetting, were excluded from this calculation to ensure comparability.

### Statistical analysis

If the testing result was reported as IU/mL, it was converted to copies/mL using the manufacturer’s conversion (1 copy = 1.71 IU, 1 IU = 0.584 copies). All VL results were transformed to log10 copies/mL for further statistical analysis.

VL results reported as TND (target not detected) were defined as amplification negative; otherwise, they were defined as amplification positive. The quantities of positive and negative samples, according to the amplification result, were separately counted for each of the three assays. Next, the positive percent agreement (PPA) and overall percent agreement (OPA) between the evaluated and reference assays were compared to assess qualitative agreement. Cohen’s kappa (κ) was used to assess agreement between each evaluated assay and the reference assay. The numerical values of VL, expressed in log10 copies/mL, were analyzed for consistency within the linear quantitative range in both the evaluated and reference assays. For the stratified analysis of various VL levels, three concentration gradients were delineated on the basis of the VL results obtained from the reference assay: low (20–1000 copies/mL), medium (1000–100,000 copies/mL), and high (≥100,000 copies/mL). Low levels were further subdivided into two groups: subgroup 1 (20–200 copies/mL) and subgroup 2 (200–1000 copies/mL).

Deming linear regression was used to calculate slope, intercept, and Pearson correlation coefficient (r) values between the evaluated HIV-1 VL assays (Livzon or SymBio) and the reference assay (Roche) via OriginPro 2018C (OriginLab Corp., Northampton, MA, USA). Assuming equal measurement errors for both assays, the variance ratio λ was set to 1. Bland‒Altman analysis was performed to evaluate the agreement between the assays, with the results processed via MedCalc v19.7.2 (MedCalc Software Co. Ltd., Ostend, Belgium). Differences between the two domestic assays (Livzon vs. SymBio) were assessed using a paired t-test.

## Results

### Sample profile

A total of 279 eligible samples were included, and the HIV-1 VL assays were conducted. All the samples were from patients with HIV-1, of which 140 (50.2%) were treated with ART and the remaining 139 (49.8%) were ART-naive.

### Concordance of qualitative results

Qualitative concordance between the Livzon and SymBio HIV-1 VL assays and the Roche reference assay was assessed. As shown in Table 1, both the Livzon and SymBio assays yielded identical numbers of samples with positive or negative amplification. The positive percentage agreement between the Livzon (or SymBio) and Roche assays was 98.06% (95% confidence interval [CI]: 95.54–99.17%), and the overall agreement was 94.26% (95% CI: 90.89–96.44%), with a *kappa* value of 0.53 (95% CI: 0.32–0.73).

**Table 1:** Qualitative concordant samples between the reference HIV-1 VL assay and the evaluated assays.

Evaluated assays	Reference assay		Total
	Positive	Negative	
Positive	253	11	264
Negative	5	10	15
Total	258	21	279

### Concordance of the quantitative results from the Livzon assay

**Fig 1:**
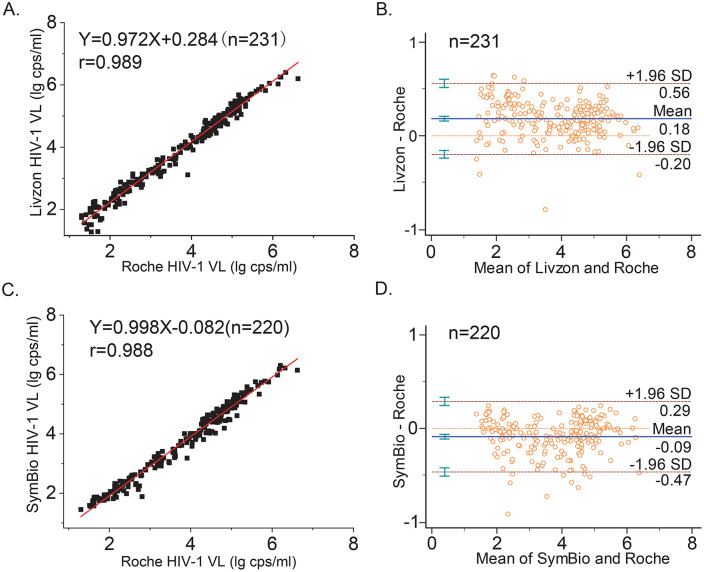
Quantitative results for clinical samples between the reference HIV-1 VL assay and the evaluated assays (Livzon or SymBio). Pearson correlation plot (A) and Bland–Altman scatter plot (B) between the Roche HIV-1 VL assay and the Livzon assay for all 231 quantifiable samples. Pearson correlation plot (C) and Bland–Altman scatter plot (D) between the Roche HIV-1 VL assay and the SymBio assay for all 220 quantifiable samples. The Bland–Altman scatter plot illustrates the differences between the two assays against their averages. The solid blue line represents the mean bias, whereas the dashed red lines delineate the limits of statistically acceptable bias, which are defined as the mean bias ± 1.96 standard deviations (SDs) of bias.

A quantitative concordance analysis was performed on 231 samples whose VLs fell within the linear quantitative ranges of both the Livzon and Roche assays. Linear regression indicated a strong correlation between the Livzon and Roche assays (r = 0.989, *P* < 0.001; Fig 1A). The Bland‒Altman scatter plot revealed a mean bias of 0.18 log10 copies/mL, with the Livzon assay showing slightly higher values than the Roche assay on an overall average (Fig 1B). A total of 96.54% (223/231) of the paired VLs fell within the 95% CI for the limits of agreement (LoA: –0.20 to 0.56 log10 copies/mL) (Fig 1B).

**Fig 2:**
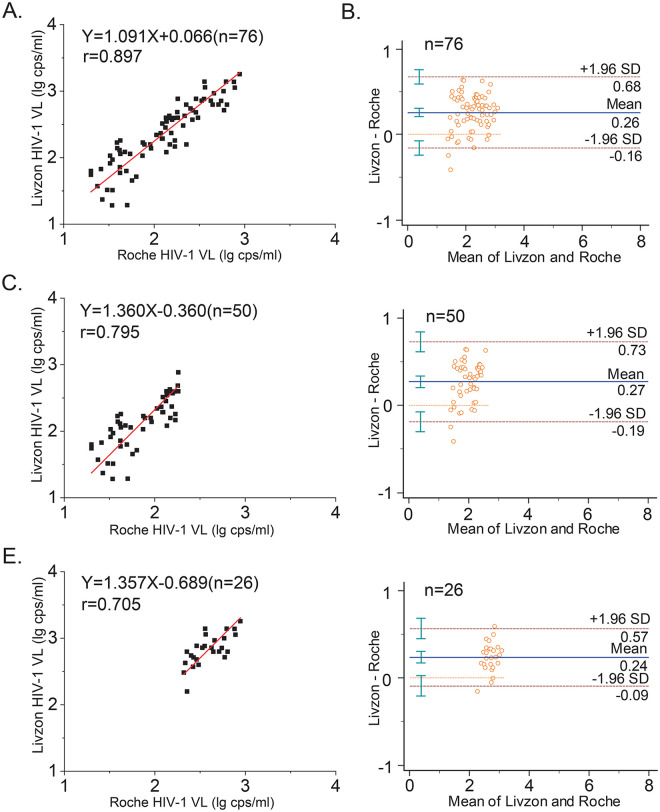
Comparison of HIV-low VL results between the Roche and Livzon assays. Pearson correlation plots for samples with 20 copies/mL ≤ VL < 1000 copies/mL (A), 20 copies/mL ≤ VL < 200 copies/mL (C), and 200 copies/mL ≤ VL < 1000 copies/mL (E). Bland–Altman scatter plots for samples with 20 copies/mL ≤ VL < 1000 copies/mL (B), 20 copies/mL ≤ VL < 200 copies/mL (D), and 200 copies/mL ≤ VL < 1000 copies/mL (F).

The Pearson correlation analysis revealed significant associations between the two assays (Livzon and Roche assays) of the samples stratified by the VL levels. Specifically, the r values for samples with low (20–1000 copies/mL, n = 76), medium (1000–100,000 copies/mL, n = 115), and high (≥100,000 copies/mL, n = 40) VL levels were 0.897 (Fig 2A), 0.960 and 0.906, respectively. The Bland‒Altman analysis demonstrated that the mean biases (Livzon–Roche) for the samples with low, medium and high levels were 0.26 log10 copies/mL (Fig 2B), 0.14 log10 copies/mL and 0.14 log10 copies/mL, respectively. For the 20–200 copies/mL subgroup (n = 50), the r for the two assays was 0.795 (Fig 2C), and the 95% CI for the LoA was –0.19 to 0.73 log10 copies/mL from the Bland‒Altman analysis (Fig 2D). For the 200–1000 copies/mL subgroup (n = 26), the r for the two assays was 0.705 (Fig 2E), and the 95% CI for the LoA was –0.09 to 0.57 log10 copies/mL from the Bland‒Altman analysis (Fig 2F).

### Concordance of the quantitative results from the SymBio assay

**Fig 3:**
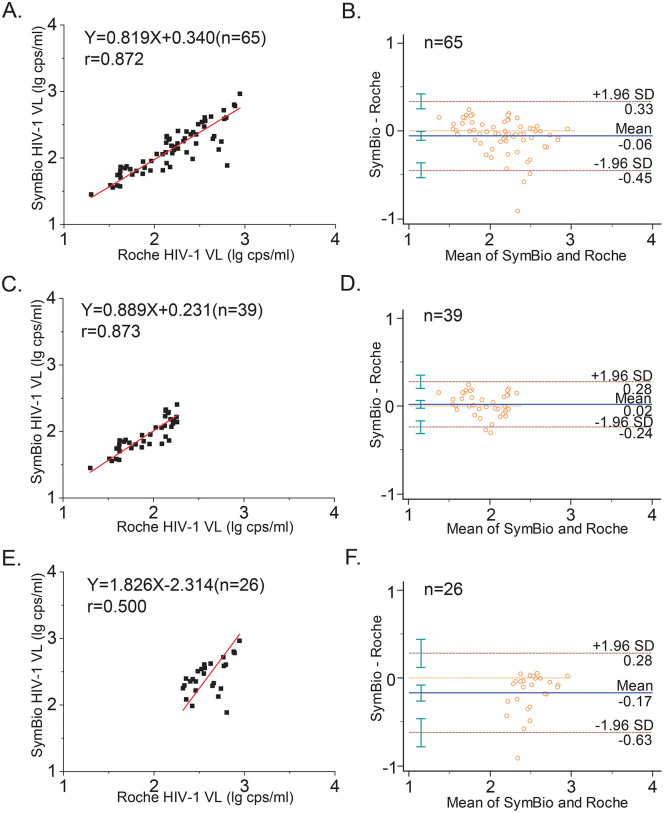
Comparison of HIV-low VL results between the Roche and SymBio assays. Pearson correlation plots for samples with 20 copies/mL ≤ VL < 1000 copies/mL (A), 20 copies/mL ≤ VL < 200 copies/mL (C), and 200 copies/mL ≤ VL < 1000 copies/mL (E). Bland–Altman scatter plots for samples with 20 copies/mL ≤ VL < 1000 copies/mL (B), 20 copies/mL ≤ VL < 200 copies/mL (D), and 200 copies/mL ≤ VL < 1000 copies/mL (F).

Using 220 samples with both the SymBio and Roche VLs within their linear ranges, we conducted a quantitative concordance analysis. The results revealed a strong linear correlation between the two VL assays (r = 0.988, *P* < 0.001) (Fig 1C). Bland‒Altman analysis showed a mean bias of −0.09 log10 copies/mL, with SymBio yielding slightly lower values than Roche on average (Fig 1D). A total of 95.45% (210/220) of the paired VLs fell within the 95% CI for the LoA (−0.47 to 0.29 log10 copies/mL) (Fig 1D). The r values for samples with low (n = 65), medium (n = 115), and high (n = 40) VL levels were 0.872 (Fig 3A), 0.947 and 0.924, respectively. Bland‒Altman analysis revealed that the mean biases (SymBio–Roche) for the samples with low, medium and high VL levels were −0.06 log10 copies/mL (Fig 3B), −0.12 log10 copies/mL and −0.05 log10 copies/mL, respectively. In the subgroup with a concentration range of 20 − 200 copies/mL (n = 39), the r between the two assays was 0.873 (Fig 3C), with a 95% CI for the LoA from the Bland‒Altman analysis ranging from −0.24 to 0.28 log10 copies/mL (Fig 3D). For the 200–1000 copies/mL subgroup (n = 26), the r between the two assays was 0.500 (Fig 3E), with a 95% CI for the LoA from the Bland‒Altman analysis ranging from −0.63 to 0.28 log10 copies/mL (Fig 3F).

### Comparison between Livzon and SymBio assays

**Fig 4:**
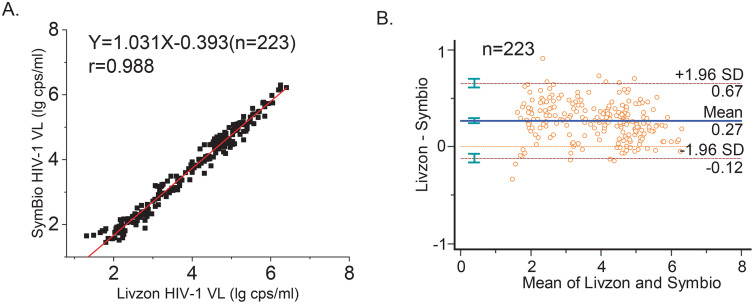
Quantitative results for clinical samples between the Livzon and SymBio assays. Pearson correlation plot (A) and Bland–Altman scatter plot (B) between the Livzon HIV-1 VL assay and the SymBio assay for all 223 quantifiable samples.

To evaluate the consistency between the two domestic assays, a head-to-head comparison was performed on 223 samples quantifiable by both methods. Deming regression showed a slope of 1.031 and an intercept of −0.393 (r = 0.988) (Fig 4A). The mean difference (Livzon – SymBio) was 0.27 log10 copies/mL. A paired t-test indicated a significant difference between the two assays (*P* < 0.001). A total of 95.52% (213/223) of the paired VLs fell within the 95% CI for the limits of agreement (LoA: –0.12 to 0.67 log10 copies/mL) from the Bland‒Altman analysis (Fig 4B).

### Analysis of discrepant results

As presented in Table 2, nine samples identified as TND by the Roche assay were detected with quantifiable viral loads by the Livzon assay, ranging from 18.1 copies/mL to 66.9 copies/mL. Additionally, two samples were detected as amplification positive but not accurately quantifiable by the Livzon assay (samples 28 and 167, < 17.6 copies/mL). The SymBio assay showed similar discrepancies with the Roche assay. Specifically, there were eleven samples for which the Roche assay reported TND, whereas the SymBio assay detected viral loads below 29.4 copies/mL. However, six samples that had detectable but unquantifiable viral loads (<20 copies/mL) in the Roche assay were not detected by at least one of the evaluated assays.

**Table 2:** Samples with discordant qualitative results between the reference HIV-1 VL assay and the evaluated assays.

Sample ID	Subtypes	Roche(copies/mL)	Livzon(copies/mL)	SymBio(copies/mL)
20	ND	TND	45.4	TND
23	ND	TND	19.3	TND
24	ND	TND	TND	<29.2
26	ND	TND	34.9	<29.2
28	ND	TND	<17.5	<29.2
35	CRF07_BC	TND	TND	<29.2
40	ND	TND	18.1	<29.2
41	CRF07_BC	TND	TND	<29.2
42	CRF01_AE	TND	TND	<29.2
65	CRF07_BC	TND	55.3	<29.2
75	ND	TND	37.3	TND
136	ND	TND	42.5	TND
167	CRF01_AE	TND	<17.5	<29.2
182	ND	TND	59.1	<29.2
257	CRF07_BC	TND	66.9	<29.2
31	CRF07_BC	<20	TND	TND
32	CRF01_AE	<20	TND	TND
43	CRF01_AE	<20	<17.5	TND
68	CRF08_BC	<20	TND	<29.2
70	CRF08_BC	28.9	TND	<29.2
72	CRF07_BC	<20	30.6	TND
129	CRF07_BC	<20	TND	TND

The results of the evaluated HIV-1 VL assays (Livzon or SymBio) that are discordant with the Roche assay are highlighted in gray. Abbreviations: ND: not detected (due to failed amplification); TND: target not detected.

As presented in Table 3, the gray-shaded samples highlight significant discrepancies in the VL results between the reference Roche assay and the evaluated assays (Livzon and SymBio), defined as differences greater than 0.5 log10 copies/mL. Specifically, the Livzon assay detected higher VLs in eight samples, with differences (Livzon‒Roche) ranging from 0.51 to 0.64 log10 copies/mL. Conversely, the SymBio assay exhibited discrepancies in the opposite direction for seven samples, with differences (SymBio‒Roche) ranging from −0.92 to −0.51 log10 copies/mL. Notably, for Sample 135, both evaluated assays yielded significantly lower VLs than Roche (Livzon: −0.80 log10 copies/mL; SymBio: −0.73 log10 copies/mL).

**Table 3:** Differentiated HIV-1 VL results between the reference assay and the evaluated assays.

Sample ID	Subtypes	Roche		Livzon			SymBio		
		copies/mL	log10 copies/mL	copies/mL	log10 copies/mL	Livzon-Roche (log10 copies/mL)	copies/mL	log10 copies/mL	SymBio-Roche (log10 copies/mL)
4	CRF07_BC	22200	4.35	71700	4.86	0.51	32591	4.51	0.17
77	CRF01_AE	514	2.71	630	2.80	0.09	134	2.13	−0.58
125	ND	39	1.59	169	2.23	0.64	39	1.59	0.00
131	CRF07_BC	39	1.59	139	2.14	0.55	55	1.74	0.15
134	CRF07_BC	33	1.51	107	2.03	0.52	39	1.59	0.08
135	CRF01_AE	8110	3.91	1300	3.11	−0.80	1521	3.18	−0.73
144	CRF07_BC	182	2.26	767	2.88	0.62	255	2.41	0.15
165	CRF01_AE	360	2.56	1390	3.14	0.59	406	2.61	0.05
169	CRF07_BC	42	1.63	181	2.26	0.63	51	1.70	0.08
174	CRF01_AE	20600	4.31	30500	4.48	0.17	6421	3.81	−0.51
203	CRF07_BC	145000	5.16	479000	5.68	0.52	201492	5.30	0.14
225	CRF01_AE	66900	4.83	71200	4.85	0.03	15509	4.19	−0.63
226	CRF01_AE	137000	5.14	204000	5.31	0.17	41047	4.61	−0.52
233	CRF01_AE	40500	4.61	28400	4.45	−0.15	10608	4.03	−0.58
258	CRF01_AE	637	2.80	626	2.80	−0.01	77	1.89	−0.92

The results of the evaluated HIV-1 VL assays (Livzon or SymBio) that differ from the Roche assay by more than 0.5 log10 copies/mL are highlighted in gray.

Abbreviations: ND: not detected (due to failed amplification).

### Assay comparison in the context of diverse subtypes

HIV genotyping results were obtained from 253 samples. Diverse subtypes were identified, among which CRF07_BC (122, 48.2%) and CRF01_AE (90, 35.6%) were predominant, followed by CRF08_BC (18, 7.1%), B (3, 1.2%), CRF55_01B (3, 1.2%), CRF64_BC (3, 1.2%), CRF85_BC (3, 1.2%), CRF59_01B (2, 0.8%), A1 (1, 0.4%), C (1, 0.4%), CRF65_cpx (1, 0.4%), and CRF67_01B (1, 0.4%). A total of 5 samples were classified as unique recombinant forms (URFs) (2.0%), of which 3 samples displayed a recombinant genomic structure of CRF01_AE and CRF07_BC, one sample was URF(B/C), and the remaining sample was CRF01_AE/B. Subtypes were classified into three groups: CRF01_AE, CRF07_BC, and other subtypes (including all remaining subtypes and URFs) for further quantitative comparisons.

**Fig 5:**
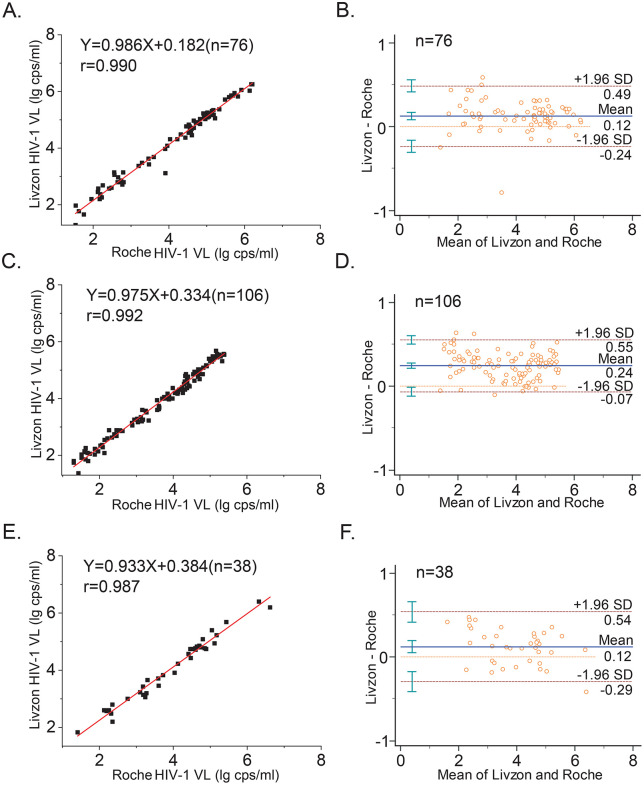
Comparison of HIV VL results between the Roche and Livzon assays. Pearson correlation plots for samples with CRF01_AE (A), CRF07_BC (C) and other subtypes (E). Bland–Altman scatter plots for samples with CRF01_AE (B), CRF07_BC (D) and other subtypes (F).

Among samples with known HIV-1 subtypes, Pearson correlations between the Livzon and Roche assays were compared; r values for CRF01_AE (n = 76), CRF07_BC (n = 106), and other subtypes (n = 38) were 0.990 (Fig 5A), 0.992 (Fig 5C), and 0.987 (Fig 5E), respectively. Bland‒Altman analysis revealed that the mean biases (Livzon − Roche) for the subtypes CRF01_AE, CRF07_BC and other subtypes were 0.12 log10 copies/mL (Fig 5B), 0.24 log10 copies/mL (Fig 5D) and 0.12 log10 copies/mL (Fig 5F), respectively. In total, 94.74% (72/76) of the samples with subtype CRF01_AE fell within the 95% CI for the LoA (−0.24 to 0.49 log10 copies/mL) (Fig 5B), 97.17% (103/106) of the samples with subtype CRF07_BC fell within the 95% CI for the LoA (−0.07 to 0.55 log10 copies/mL) (Fig 5D), and 97.37% (37/38) of the samples with other subtypes fell within the 95% CI for the LoA (−0.29 to 0.54 log10 copies/mL) (Fig 5F).

**Fig 6:**
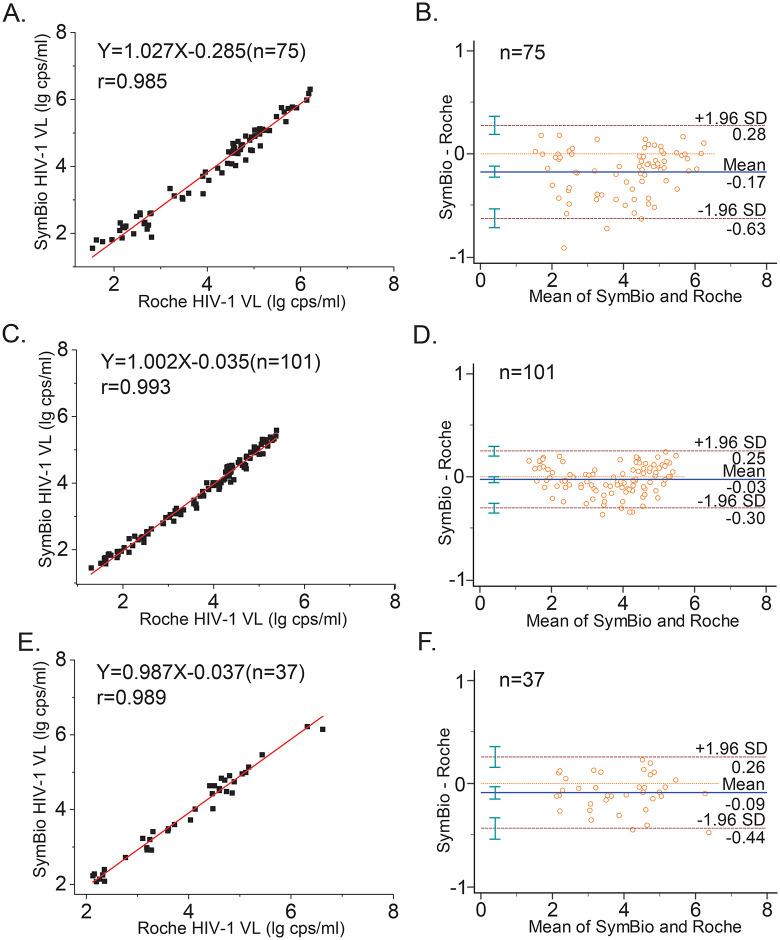
Comparison of HIV VL results between the Roche and SymBio assays. Pearson correlation plots for samples with CRF01_AE (A), CRF07_BC (C) and other subtypes (E). Bland–Altman scatter plots for samples with CRF01_AE (B), CRF07_BC (D) and other subtypes (F).

The r values for the two assays (SymBio vs Roche) with samples of subtypes CRF01_AE (n = 75), CRF07_BC (n = 101), and other subtypes (n = 37) were 0.985 (Fig 6A), 0.993 (Fig 6C) and 0.989 (Fig 6E), respectively. Based on the Bland‒Altman analysis, the mean biases (SymBio − Roche) for CRF01_AE, CRF07_BC, and other subtypes were −0.17 log10 copies/mL (Fig 6B), −0.03 log10 copies/mL (Fig 6D), and −0.09 log10 copies/mL (Fig 6F), respectively. Overall, 96.00% (72/75) of the samples with subtype CRF01_AE (Fig 6B), 97.03%(98/101) of the samples with subtype CRF07_BC (Fig 6D), and 94.59%(35/37) of the samples with other subtypes (Fig 6F) fell within the 95% confidence LoAs (−0.63 to 0.28 log10 copies/mL, −0.30 to 0.25 log10 copies/mL, and −0.44 to 0.26 log10 copies/mL, respectively).

### Evaluation of cost and turnaround time (TAT)

Compared with the Roche assay, the domestic assays (Livzon and SymBio) were priced more than 30% lower. Regarding TAT for a standard 96-test batch, the Roche COBAS assay required 9.3 hours, whereas the Livzon assay completed testing within 4.6 hours and the SymBio assay within 7.5 hours. Thus, the domestic assays achieved a 20–50% reduction in overall assay time compared to the Roche reference.

## Discussion

In this study, we compared two Chinese HIV-1 VL assays with the Roche COBAS AmpliPrep/COBAS TaqMan HIV-1 Test v2.0 using clinical samples. To minimize RNA degradation, all assays were performed concurrently on freshly processed samples. The r values between the reference HIV-1 VL assay and the Livzon/SymBio assay for the quantitative results were 0.989 and 0.988, respectively. The average biases in the two groups of comparison tests were 0.18 log10 copies/mL and −0.09 log10 copies/mL. Overall, the domestic assays showed moderate qualitative agreement (κ = 0.53) with the Roche assay. This Kappa value was likely constrained by the ‘Kappa paradox’ due to high sample prevalence and the inclusion of specimens near the detection limit. Nevertheless, the high PPA (>98%) and strong quantitative correlations confirm their suitability for clinical HIV-1 monitoring.

Accurate detection of low viral loads is crucial for assessing ART success, as virological failure is defined by VL results below 200 or 1000 copies/mL. Additionally, achieving the goal of VL suppression to undetectable levels, or less than 50 copies/mL, is challenging, and the occurrence of viral blips (50 − 400 or 50 − 1000 copies/mL) during ART is associated with an increased risk of subsequent virologic failure [11]. Such results may be mistaken for treatment failure, potentially leading to unnecessary medication changes [12]. This study evaluated the performance of the two domestic assays in the low VL group (20 − 1000 copies/mL). The r values between the reference HIV-1 VL assay and the Livzon assay or SymBio assay for quantitative results were 0.897 and 0.872, respectively, suggesting the strong quantitative detection capabilities of these two domestic assays at low VL levels. It is anticipated that these two domestic assays will enable precise detection at low viraemia levels, facilitating timely and efficient treatment for patients with HIV/AIDS undergoing ART. Additionally, a non-uniform proportional bias was observed, with greater variance at lower viral loads. While reliable for identifying treatment failure (>1,000 copies/mL), absolute values should be interpreted cautiously when monitoring low-level viremia due to increased measurement uncertainty near the detection limit. Discrepancies between the reference assay and the domestic assays in low-level RNA samples may be due to random variations within each assay’s detection limit [13,14].

Compared with the COBAS assay, the Livzon assay demonstrated a higher VL value, with a mean bias of 0.18 log10 copies/mL, consistent with previous studies [15]. In the low-VL group, the Livzon assay exhibited a higher mean bias of 0.26 log10 copies/mL. Conversely, the SymBio assay yielded a lower VL value than the COBAS assay, with a mean bias of −0.09 log10 copies/mL. The mean bias remained relatively stable across low (20 − 1000 copies/mL, mean bias: −0.06 log10 copies/mL), medium (1000 − 100,000 copies/mL, mean bias: −0.12 log10 copies/mL), and high (≥100,000 copies/mL, mean bias: −0.05 log10 copies/mL) VL levels. Several studies have highlighted discrepancies in HIV-1 RNA quantification across various commercial HIV-1 VL assays [12–18]. The World Health Organization (WHO) International Standards are widely recognized as authoritative global standards for the calibration and characterization of quantitative molecular viral assays [19]. Different standards and calibration methods can cause discrepancies in absolute values across assays, affecting result accuracy and comparability [16]. The WHO 4th International Standard (NIBSC code: 16/194) was used as a reference for the Livzon and SymBio assays. By contrast, the Roche assay is traceable to the WHO 3rd International Standard (NIBSC code: 10/152). Reagent manufacturers frequently use secondary or tertiary standards in their production process [18], and it has been previously demonstrated that secondary materials do not always contain the same number of target copies for a given nominal value [20]. Consequently, laboratories may standardize their results against different reference standards [18]. Differences in the use of traceability standards partly explain the discrepancies in the quantity values of different assays. In addition, the design of reagents from various manufacturers results in differences in system optimization and substance interference, leading to varying amplification results for the same sample [18]. Given the accepted tolerance of ±0.5 log10 for quantitative NAT assays [19], the results of the two evaluated assays are in good agreement with the Roche HIV-1 VL assay.

Discordant VL results were observed in several samples between the evaluated assays and the reference assay (Tables 2 and 3). A previous study [15] reported two key factors influencing HIV-1 VL evaluation and comparison: (1) sample retention (RNA degradation may occur due to freeze-thaw cycles) and (2) heterogeneity in plasma aliquots (intrinsic VL differences may be introduced during testing). However, precautions were implemented to mitigate the impact of these factors during the evaluation. Fresh samples were used for concurrent testing, with aliquots recombined and thoroughly mixed prior to sampling to ensure uniformity. Previous studies have reported mismatch mutations in the amplicon regions of the HIV-1 VL assay for new HIV-1 variants, resulting in sample underquantification or false-negative test results [21]. The target regions designed by different manufacturers vary, and this variation may lead to discrepancies in detection results due to gene sequence mutations [15–17]. Inadequate binding of PCR primers and fluorescence probes to target regions may result in inconsistent amplification efficiency, leading to variations in VL measurements. Furthermore, most discrepancies observed in the samples with discordant qualitative results were clustered below 50 copies/mL. Although limited sample volumes precluded repeated precision testing, these minor variations are consistent with the Poisson distribution effect inherent in low-copy NAT assays rather than assay failure. Given that clinical thresholds for treatment failure are typically ≥200 copies/mL, these low-level fluctuations have minimal impact on routine patient management.

The highly diverse subtypes of HIV pose a unique challenge for VL testing [22], particularly in China where this diversity is significantly pronounced [23]. According to the fifth national HIV molecular epidemiological survey in 2023, at least 30 subtypes are prevalent in China. Among these subtypes, CRF07_BC and CRF01_AE are the dominant subtypes, accounting for approximately 70% of all cases (unpublished national survey data, 2023). We included 12 subtypes and 5 URFs in the clinical samples used in this study, which effectively cover the main prevalent HIV subtypes in China. A previous study [24] revealed that the distribution of subtypes observed in Zhejiang was similar to that of China as a whole, suggesting that the results of this study may reflect the HIV variations existing in China. The r values between the reference HIV-1 VL assay and the two evaluated assays ranged from 0.985 to 0.993 for the different subtypes (CRF01_AE, CRF07_BC and other subtypes). This suggests that the selected target gene regions for detection are relatively conserved, demonstrating their adaptability and suitability for use in regions with high genetic diversity of HIV strains. Concerns regarding target selection in the pol gene affecting quantification in patients with drug-resistance mutations were addressed by the assay design. The Livzon and SymBio assays target highly conserved regions within pol (specifically avoiding common mutation hotspots in PR/RT) and combine this with a dual-target strategy (including LTR or gag). This redundancy ensures that even if mutations compromise amplification in one region, quantification remains accurate. Notably, consistent with their NMPA-certified LoDs (<20 copies/mL), both Livzon and SymBio assays achieved a 100% detection rate (200/200) for all samples with viral loads >100 copies/mL as determined by the reference Roche assay. This confirms that both assays reliably meet the WHO-recommended threshold for viral load monitoring in resource-limited settings.

Given the considerable variability of HIV, continuous surveillance of its genetic diversity is critical, along with careful consideration of the influence of NAT assays. Furthermore, further research on the performance of these assays in diverse subtypes can improve the accuracy of VL testing and enhance HIV/AIDS patient management.

Beyond analytical performance, practical considerations such as cost and TAT are critical for the implementation of viral load testing in resource-limited settings. In our evaluation, both domestic assays demonstrated clear operational advantages: reagent costs were reduced by over 30%, and batch TATs were shortened by 20–50% compared with the Roche CAP/CTM system. These improvements have significant implications for scaling up routine monitoring, as reduced costs can facilitate broader access to testing, and shorter TATs may enable faster clinical decision-making and improved patient management. These advantages, coupled with comparable analytical accuracy, underscore the potential of domestically manufactured assays to bridge the monitoring gap in low-resource settings. It should be noted, however, that the CAP/CTM reference platform is being progressively phased out and replaced by fully automated, high-throughput systems such as the cobas 6800/8800; therefore, the TAT and cost comparisons presented here should be interpreted with caution in the context of this ongoing platform transition.

Several limitations warrant consideration. First, as a clinical performance evaluation of NMPA-approved products, independent re-verification of the LoD and extensive specificity testing (e.g., with HBV/HCV) were not repeated, as these were established during regulatory certification. Second, although a commercial WHO genotype panel was not used, the 279 clinical samples covering 12 subtypes provide robust “real-world” evidence of inclusivity. Third, despite the emergence of newer platforms like the Cobas 6800/8800, we selected the CAP/CTM v2.0 as it best reflects the current diagnostic landscape and routine practices in the majority of Chinese healthcare facilities. Finally, sample volume constraints precluded re-testing of discrepancies near the LoD; however, given their WHO-standard calibration, these may represent enhanced sensitivity for low-level viremia.

Our findings indicate that the two Chinese-manufactured HIV-1 VL assays demonstrate favorable performance characteristics, including sensitivity, precision, and reliability. In addition, these assays offer a cost-effective solution and improved efficiency, with reductions in both reagent costs and turnaround time. These assays constitute valuable additions to the HIV molecular diagnostic toolkit and could expand access to accurate and affordable VL testing, particularly in resource-limited settings.
